# A Multi-Year Examination of Gardening Experience and Fruit and Vegetable Intake During College

**DOI:** 10.3390/nu11092088

**Published:** 2019-09-04

**Authors:** Daniel Staub, Sarah E. Colby, Melissa D. Olfert, Kendra Kattelmann, Wenjun Zhou, Tanya M. Horacek, Geoffrey W. Greene, Ivana Radosavljevic, Lisa Franzen-Castle, Anne E. Mathews

**Affiliations:** 1 Food Science and Human Nutrition Department, University of Florida, Gainesville, FL 32611-0370, USA; 2 Department of Nutrition, College of Education, Health & Human Sciences, University of Tennessee, Knoxville, TN 37996, USA; 3 Human Nutrition and Foods in Animal and Nutritional Sciences Division, Davis College of Agriculture, Natural Resources and Design, West Virginia University, Morgantown, WV 26506-6108, USA; 4 Department of Health and Nutritional Sciences, South Dakota State University, Brookings, SD 57007, USA; 5 Department of Business Analytics & Statistics, University of Tennessee Knoxville, Knoxville, TN 37996-0532, USA; 6 Department of Public Health, Food Studies and Nutrition, Syracuse University, Syracuse, NY 13244, USA; 7 Department of Nutrition and Food Sciences, University of Rhode Island, Kingston, RI 02881, USA; 8 Nutrition and Health Sciences Department, University of Nebraska-Lincoln, Lincoln, NE 68583-0806, USA

**Keywords:** gardening, fruit and vegetable intake, college, childhood, long-term

## Abstract

Gardening has been positively associated with fruit and vegetable (FV) consumption based on short-term studies among children, but long-term data among adolescents and young adults are lacking. This investigation sought to elucidate the association between gardening experience and FV intake among college students over a two-year period. Students (*N* = 593) from eight universities were assessed at the end of their freshman (Y1) and sophomore (Y2) years during the springs of 2016 and 2017, respectively. At each time point, participants completed the NCI FV Screener and questions related to gardening experience and FV-related attitudes and behaviors. Students were then categorized into four groups based on gardening experience: Gardened only during the first or second year (Y1 only and Y2 only gardeners), gardened both years (Y1+Y2 gardeners), and non-gardeners. While both Y1 only and Y1+Y2 gardeners reported significantly higher FV intake relative to non-gardeners at Y1 (2.3 ± 0.9 and 2.6 ± 0.7 versus 1.9 ± 0.6 cup equivalents (CE)/day, respectively; *p* < 0.01), only Y1+Y2 gardeners differed from non-gardeners at Y2 (2.4 ± 0.6 versus 1.8 ± 0.5 CE/day; *p* < 0.001). Additionally, Y1+Y2 gardeners reported more frequent engagement of several FV-related behaviors, including shopping at farmers’ markets, eating locally grown foods, and cooking from basic ingredients; and were five times more likely to have gardened during childhood (OR: 5.2, 95%, CI: 3.5–8.8; *p* < 0.001). Findings suggest that while isolated gardening experiences during college are associated with FV intake, reoccurring experience may be essential for sustained benefit.

## 1. Introduction

The association between fruit and vegetable (FV) intake and reduced risk of many chronic diseases is among the most well characterized and universally recognized aspects of the relationship between dietary patterns and health. Virtually every major health organization emphasizes the importance of a diet rich in FV for chronic disease prevention and recommends the inclusion of a variety of FV as part of a healthy eating pattern throughout the lifecycle [1,2]. Nevertheless, average FV consumption in the U.S. has failed to meet national guidelines for decades, and currently less than one fifth of Americans meet recommendations [3]. This pervasive issue has heavily implicated the rising prevalence of chronic disease over the past several decades [1]. 

From a public health perspective, strategies to address suboptimal FV intake during early adulthood may be particularly advantageous. Emerging adulthood is not only a period of physical maturation, but also a time of psychosocial maturation during which many lifestyle behaviors are developed and solidified. As such, health behaviors among emerging adults may be more amenable to promotional strategies compared to those of older adults [4]. Furthermore, behaviors developed during adolescence and early adulthood frequently persist into late adulthood [5]. Hence, efforts to promote FV intake during the transition to adulthood may have a lasting impact that could eventually contribute to a reduction in chronic disease. However, despite an extensive body of research in this area, effective approaches to FV promotion are limited. Common strategies, such as nutrition education, social marketing, and food environment modification, have been implemented in a variety of contexts, yet successes remain limited and generally modest [6,7,8].

Gardening is a less conventional approach to promoting FV intake that has generated interest in the nutrition and public health field in recent years. In addition to the opportunity to incorporate lessons in nutrition and food preparation, gardening programs provide valuable exposure to FV. Gardening interventions in school and community settings have demonstrated success among school-aged children and adolescents [9,10,11,12,13,14,15]. In a 2017 review of gardening interventions to increase FV consumption among children [16], the authors determined that, of the 14 articles reviewed, 10 reported statistically significant increases in FV consumption in the post-intervention period. However, the authors noted that improvements in FV consumption tend to be modest. Other surveys of the literature have resulted in less consist findings. In another recent review [17] Ohly et al. identified 13 studies that examined the impact of school gardens on children’s FV intake, only two of which reported statistically significant increases. Investigators also noted that these were among the few studies in this area to explicitly measure FV intake as opposed to solely examining behavioral mediators of FV intake. While reports of FV knowledge, attitudes, and preferences have been fairly compelling [18,19,20], these measures do not necessarily represent actual improvements in FV intake. For example, in the review by Ohly et al. [17], the authors concluded that school gardens increase willingness to try and preferences for FV among school children, but more robust quantitative research is needed to substantiate any further conclusions. 

Another limitation of the current gardening literature is the lack of long-term data. Most investigations have been limited to a single period of exposure, often spanning one academic year, with short follow-up periods or no follow-up at all [9,10,12,14]. Of the few investigations extending beyond one year, reported outcomes have been markedly positive and far more consistent [21,22,23]. For example, a prospective study by Wang et al. evaluated 4th and 5th graders in a mid-sized school district to determine the effect of a multi-faceted school-based intervention on students’ FV intake over two years [21]. Researchers found that those with the greatest exposure to the intervention increased FV intake by 0.5 cups/day, whereas those with little to no exposure decreased FV intake by 0.3 cups/day (*p* < 0.01). However, as with many interventions, this program was not limited to gardening and included school lunch enhancement initiatives, cooking classes, and other experiential learning programs. In another long-term study spanning three growing seasons, Castro et al. evaluated changes in FV intake among a group of 95 children and adolescents aged 2–15 years in response to a community-based health intervention [22]. Participants and their families engaged in weekly gardening sessions from April through November over each of the three program years. At the end of the program, investigators observed a 2 serving/week increase in fruits (*p* < 0.001) and 5 serving/week increase in vegetables (*p* < 0.001) among this group of children and adolescents. Although gardening was the core component of the intervention, this program also included a 7–week cooking and nutrition workshop, as well as social activities and events. Furthermore, the observed changes in FV intake were based on parental reports of the children’s dietary intake. Despite some limitations in terms of applicability in the present context, these long-term investigations provide compelling evidence to suggest that duration and consistency may be key to achieving meaningful improvements in FV intake in response to gardening. 

Our previous cross-sectional investigation of 1121 college freshmen from eight universities revealed that students with childhood and recent gardening experience reported significantly greater FV intake compared to those who did not garden (2.5 ± 0.6 versus 1.9 ± 0.5 cup equivalents (CE), respectively; *p* < 0.001) [24]. This was among the few reports in a population of college students and, to our knowledge, was the first to examine gardening experiences in both childhood and young adulthood. We now look to expand upon this work using follow-up data from students in this original cohort to explore how gardening experience over one’s freshman and sophomore years relates to students’ FV intake. Specifically, our primary objective was to determine whether FV intake is associated with students’ gardening experience during their freshmen and sophomore academic years and to examine how the change in gardening experience from year to year influences this relationship. Our secondary objective was to determine how students’ gardening experience relates to other FV-related attitudes and behaviors, such as meal planning to include more FV, and to evaluate the extent to which childhood gardening predicts gardening experience in college. 

## 2. Materials and Methods 

### 2.1. Study Design

This prospective investigation was conducted during the formative stages of the Get FRUVED project, a multi-institutional project with the goal of promoting health behaviors among older adolescents. In this stage, eight U.S. universities served as partnering sites for intervention development using a community based participatory research approach in which college students, not included in this analysis, worked together to develop and pilot test intervention strategies that would later be used as part of a randomized trial on other college campuses. Here, freshmen attending these same eight universities were recruited and enrolled in late summer through early fall of 2015 as previously described [24]. All participants who completed a baseline assessment in fall 2015 were invited to attend on-site follow-up assessment visits at the end of their freshman (Y1) and sophomore (Y2) academic years. 

While the protocol for data collection is detailed elsewhere [24], briefly, students attended in-person assessments and completed a battery of lifestyle and health-related questionnaires through a secure, web-based platform. Participants also completed anthropometric measurements administered by trained researchers. For a complete case analysis, we included only those who provided complete data at both time points in the final sample. To ensure the risk of bias from missing data was minimal, a preliminary analysis was conducted to compare characteristics between those included and those excluded at each time point independently. Participating universities included Auburn University, Kansas State University, South Dakota State University, Syracuse University, University of Florida, University of Maine, University of Tennessee, and West Virginia University. All study procedures were approved by the University of Tennessee Institutional Review Board for all participating universities, except Kansas State University, Auburn University, and the University of Florida, where separate institutional approval was required. Participants provided written informed consent prior to the collection of data. The study was registered at Clinicaltrials.gov (NCT02941497).

### 2.2. Participants 

The study cohort was comprised of individuals enrolled as first-year students for the fall 2015–spring 2016 academic year at one of the eight participating universities. Students were required to be at least 18 years of age at the time of enrollment. In accordance with the design of the parent study, eligible participants were limited to students whose estimated daily FV intake was less than 2 cup equivalents (CE) of fruit and/or 3 CE of vegetables at the time of enrollment. In addition, participants were required to meet one of the additional following criteria: Having a body mass index (BMI) ≥ 25 kg/m^2^, identifying as a first-generation college student, having a parent be overweight or obese, reporting a low-income background, or identifying as a racial minority. These criteria were designed to select for students who may be predisposed to unhealthy behaviors during college for the purposes of project development.

### 2.3. Measures

#### 2.3.1. College and College Gardening Experience

At each follow-up assessment, participants completed questions related to gardening experience within the previous year. Questions were developed by investigators and reviewed by experts for content and clarity prior to establishing test-retest reliability, as described by Loso et al. [24]. Specifically, participants were asked, “In the past 12 months, how often have you been engaged in vegetable/fruit gardening activities (This can include pots on the porch, in ground garden, community garden, etc.?)” Although response options were presented as a five-item scale ranging from “Never” to “Daily,” the data were re-coded for the purposes of this analysis to dichotomize students into those with or without gardening experience in the previous year. 

Students were also asked about gardening experience during childhood. As with the previous gardening questions, items were reviewed by experts and tested for reliability. For this analysis, a single yes/no question was selected for inclusion. Specifically, participants were asked, “Growing up, did you ever participate in growing vegetables/fruits on a small or large scale (This can include pots on the porch, in ground garden, community garden, etc.?).” Again, students were dichotomized based on the presence or absence of childhood gardening experience for analysis.

#### 2.3.2. FV Intake

FV intake was determined by the National Cancer Institute (NCI) FV Screener [25], a 19-item survey validated against established instruments, such as 24-h recalls, and other food frequency questionnaires [26]. The NCI FV Screener accounts for both the frequency and quantity of FV consumed over the past 30 days to generate an average score in the form of cups per day. For the purposes of the parent study, data were converted to cup equivalents (CE) per day and are presented as such herein. 

#### 2.3.3. FV-Related Attitudes and Behaviors 

To augment measures of FV intake, questionnaires within the larger survey were screened for items assessing FV-related attitudes and behaviors. As part of the selection protocol, only items explicitly related to FV were eligible for inclusion. For example, questions such as “How often do you remind yourself to eat in moderation,” were not deemed directly relatable to FV, whereas questions such as “How often do you purposely add vegetables to meals and snacks,” were identified as directly related. Ultimately, seven items from three surveys (*Green Eating* [27], *Meal Planning* [28], and *Cooking* [29]) were included for analysis. Corresponding response options consisted of seven to nine ordinal fixed responses presented as a Likert-type scale (for assessment of attitude) or frequency scale (for assessment of behavior) (Table 3). 

#### 2.3.4. Anthropometry 

Participant BMI was calculated using measurements collected by trained research assistants using a standardized protocol [24]. Height and weight were measured in duplicate by stadiometer and digital scale, respectively, with participants’ shoes and heavy clothing removed. 

#### 2.3.5. Demographic Characteristics 

As part of each follow-up survey, students self-reported the university at which they were enrolled, as well as their sex (as assigned at birth), age, and race/ethnicity. Since participants could select all applicable response options when reporting race/ethnicity; students who identified with more than one race or ethnicity were grouped in a separate category for analysis. 

### 2.4. Data Analysis 

All data were analyzed using SAS version 9.14 statistical software. Descriptive statistics were used to characterize study participants. Cohen’s κ for yes/no gardening responses and weighted κ scores for ordinal variables were calculated to assess test–retest reliability of gardening survey questions with a separate group of undergraduate students before data collection. A linear mixed model was used to evaluate the relationship between gardening experience and FV intake at each of the two time points; i.e., end of year 1 (Y1) and end of year 2 (Y2). FV intake was analyzed on the log scale to meet the assumptions for normality, with results presented as mean ± SE. With respect to FV-related attitudes and behaviors, an ordinal regression model was constructed to compare responses between gardeners and non-gardeners, with non-gardeners serving as the reference category. The direction of each Likert or frequency scale was adjusted if necessary, so that all resulting odds ratios directly correlated with the level of agreement to each item. Lastly, binomial logistic regression was used to analyze the relationship between college gardening experience and childhood gardening experience. For each model, BMI, sex, and race/ethnicity were included as covariates with university treated as a random effect. Level of statistical significance was set at *p* < 0.05. 

## 3. Results

### 3.1. Participant Eligibility and Enrollment 

**Figure 1:**
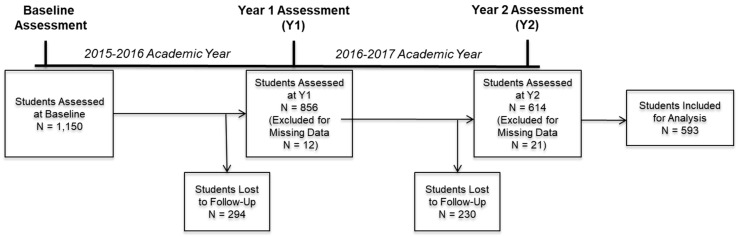
Participant enrollment and participation at baseline (Fall 2015) and each subsequent data collection timepoint, spring 2016 and spring 2017.

An overview of participant enrollment and eligibility screening over the course of the study is provided below in Figure 1. Briefly, recruitment and survey screening resulted in the identification of 2757 eligible students, of which 1150 officially enrolled in the study by attending an on-site assessment visit at the start of their freshman year. From this original cohort, a total of 856 students attended a follow-up assessment at the end of this first year (Y1). After exclusion of those with missing data, 844 students remained eligible for this analysis. Of these 844 students, 614 attended a second follow-up assessment at the end of their sophomore year (Y2). Exclusion of those with missing data at this time ultimately yielded 593 complete cases for analysis. It is worth noting that, although data from participants lost to follow-up may not be considered missing completely at random, it was confirmed that this group did not differ from the remaining sample across any of the reported measures.

### 3.2. Participant Characteristics

An overview of participant characteristics is provided in Table 1. Participants were predominately female (70.0%) and between the ages of 18 and 19 years at Y1 (30.9% and 67.1%, respectively). With respect to race and ethnicity, approximately half of students (48.7%) identified as White, while approximately one fourth (27.3%) identified as other (including biracial). The remaining sample was fairly-evenly comprised of those identifying as Black (12.6%) and Hispanic/Latino (11.1%). The distribution of students from each university ranged from about 5% to 30%, with students from Alabama (4.6%) and South Dakota (4.7%) least heavily represented and students from Florida (33.6%) most heavily represented. 

### 3.3. Prevalence of College Gardening Experience 

**Figure 2:**
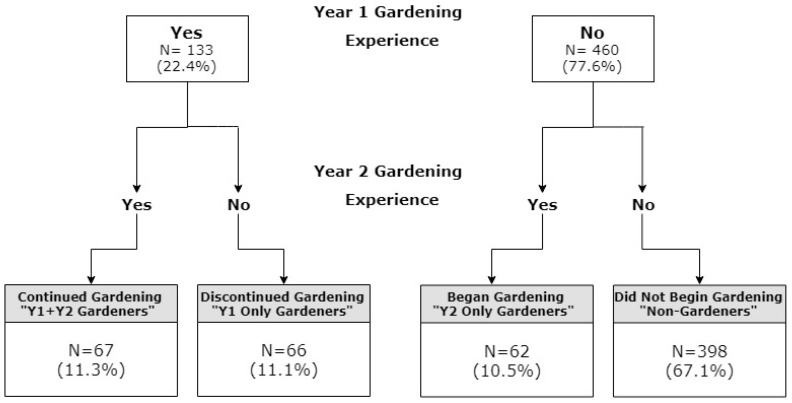
Overview of gardening experiences during the first two years of college. Students reported experiences with gardening in the previous 12 months at both year one (Y1) and Y2. Students were then grouped into one of four categories by their cumulative gardening experience over the two-year period.

Overall, the prevalence of students with gardening experience during the previous year was similar at Y1 and Y2, with approximately one in five students reporting gardening during the corresponding year (Figure 2). When gardening experience was viewed cumulatively over the two-year period, four groups of students were identified. These included (1) “non-gardeners,” or those who did not garden during either Y1 or Y2; (2) “Y1 only gardeners,” or those who gardened during Y1 but discontinued during Y2; (3) “Y2 only gardeners,” or those who did not garden during Y1 but began gardening during Y2; and (4) “Y1+Y2 gardeners,” or those who gardened during Y1 and continued gardening during Y2. With respect to the distribution of students across these groups, the majority (67.1%) were classified as non-gardeners, while the remainder was fairly evenly distributed across the other groups. 

Gardening experience varied by participant characteristics (Table 2). While there were no differences in terms of sex, significant differences were identified when students were categorized by race/ethnicity and university location. Subsequent analysis revealed that in general, gardening experience was less prevalent among both Black and Hispanic/Latino students relative to those who identified as White, and more prevalent among students from northern schools (e.g., Maine, South, and Dakota) compared to students from southern schools (e.g., Alabama or Florida). These specific differences by race/ethnicity and university location are detailed in Supplementary Tables S1 and S2, respectively.

### 3.4. FV Intake by College Gardening Experience 

**Figure 3:**
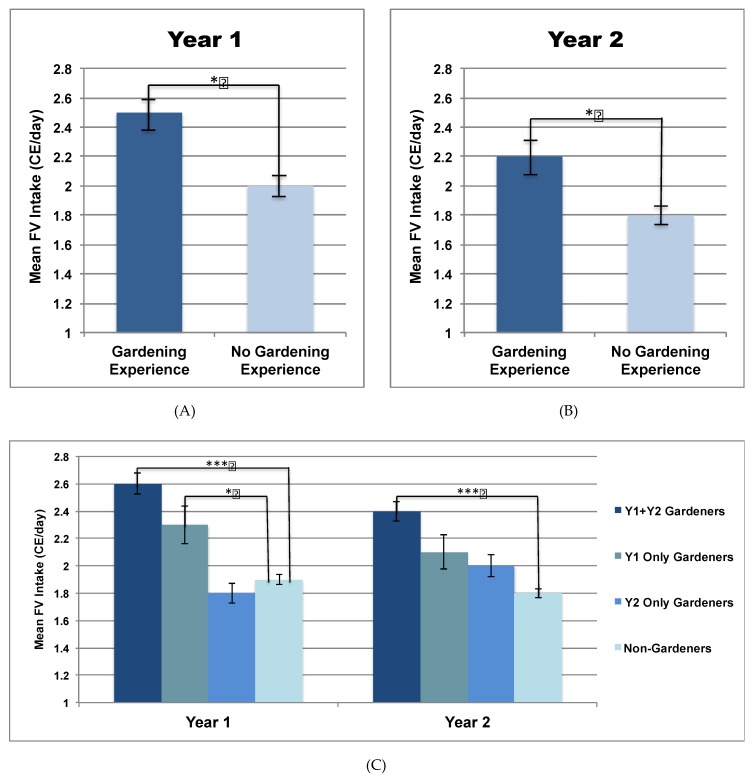
Fruit and vegetable (FV) Intake by College Gardening Experience. (**A**) Comparison of Y1 FV intake between those who did versus those who did not garden during the first year. (**B**) Comparison of Y2 FV intake between those who did versus those who did not garden during the second year (**C**) Comparison of Y1 and Y2 FV intake based on cumulative gardening experience over the two study years. BMI, sex, and race/ethnicity were included in the model as covariates with university treated as a random effect. All data are mean ± SE; * *p* < 0.05, *** *p* < 0.001.

Gardening experience in the past year was associated with higher year-end FV intake at either time point (Figure 3). More specifically, students with gardening experience during Y1 reported a FV intake 0.5 CE/day greater than their counterparts at Y1 (2.5 ± 0.4 versus 2.0 ± 0.5 CE/day; *p =* 0.02), while students with gardening experience during Y2 reported an FV intake 0.4 CE/day greater than their counterparts at Y2 (2.2 ± 0.6 versus 2.0 ± 0.4 CE/day; *p =* 0.03). With respect to cumulative gardening experience over the study period (panel C), both Y1 only gardeners and Y1+Y2 gardeners reported a higher FV intake relative to non-gardeners at Y1 (2.3 ± 0.9 and 2.6 ± 0.7 versus 1.9 ± 0.6 CE/day, respectively; *p* < 0.01). At Y2 however, a significant difference was observed only between Y1+Y2 gardeners and non-gardeners (2.4 ± 0.6 versus 1.8 ± 0.5 CE/day; *p* < 0.001). Of note, although Y2 gardeners as a whole reported significantly higher FV intake than their counterparts at Y2 (panel B), there was no significant difference in Y2 FV intake between Y2 only gardeners and non-gardeners. 

### 3.5. FV-Related Behaviors and Attitudes by College Gardening Experience

To supplement measures of FV intake and further characterize those with consistent gardening experience over the study period, FV-related attitudes and behaviors among Y1+Y2 gardeners were analyzed using non-gardeners as a reference sample. Since these groups encompass gardening experience during both Y1 and Y2, responses at both time points were analyzed to determine which attitudes and behaviors, if any, consistently differed over the years. Significant associations were identified in five of the seven items at Y1, of which four were repeatedly observed at Y2 (Table 3). Corresponding odds ratios for these items, as well as those that reached significance at only one time point, indicated that Y1+Y2 gardeners were significantly more likely to answer with a stronger level of agreement (for attitude) or higher level of frequency (for behavior) than non-gardeners. With respect to the most robust of these associations, Y1+Y2 gardeners were about three to four times more likely to eat locally grown foods at a higher frequency than non-gardeners (Y1: OR = 4.2, 95% CI: (2.5–7.0); *p* < 0.001; Y2: OR = 3.2, 95% CI: (2.0–5.3) *p* < 0.001) and about four to 4.5 times more likely to shop at farmers’ markets at a higher frequency than non-gardeners (Y1: OR = 3.9, 95% CI: (2.4–6.4); *p* < 0.001; Y2: OR = 4.7, 95% CI: (2.8–7.6); *p* < 0.001). Additionally, Y1+Y2 gardeners contemplated including FV with meals more often (Y1: OR = 1.6, 95%, CI: 1.1–2.8; *p =* 0.063; Y2: OR =1.6, 95% CI: 1.0–2.7; *p* = 0.043) and were more confident in cooking from basic ingredients (Y1: OR=2.0, 95% CI: 1.2–3.1 *p* = 0.006; Y2: OR = 1.8, 95% CI: 1.1–2.9; *p =* 0.017) across both time points. 

### 3.6. Childhood Gardening Experience as a Predictor of College Gardening Experience 

Due to a lack of long-term data, little is known about how gardening during childhood influences behaviors later in life. In light of findings from our previous work along with the present results underscoring consistency as a key differentiating factor among college students, we sought to explore the possibility that childhood gardening experience increases the propensity of students to adopt and maintain gardening experience during their college years. Accordingly, binomial logistic regression was used to determine whether Y1+Y2 gardeners were more likely to have gardened during childhood compared to non-gardeners. Our results showed that Y1+Y2 gardeners were over five times more likely to have gardened during childhood compared to non-gardeners (OR = 5.2, 95% CI: 3.5–8.8, *p* < 0.001).

## 4. Discussion

This investigation of nearly 600 college students over their first two academic years provides several unique lines of evidence to substantiate a positive association between gardening experience and FV intake among young adults. At the end of each academic year, FV intake was significantly higher among students who reported gardening during the corresponding year compared to students who did not. When students were further divided based on cumulative gardening experience over the two-year period, four unique groups were identified: Those who gardened only during the first year (Y1 only gardeners), those who gardened only during the second year (Y2 only gardeners), those who gardened during both years (Y1+Y2 gardeners), and those who did not garden during either year (non-gardeners). With respect to the primary outcome, although both Y1 only and Y1+Y2 gardeners reported significantly higher FV intake at Y1 relative to non-gardeners, only Y1+Y2 gardeners maintained a higher FV intake relative to non-gardeners at Y2. Consistent with these differences in FV intake, Y1+Y2 gardeners reported more frequent engagement in a variety of FV-related behaviors, such as meal planning to include FV, cooking from basic ingredients, and shopping at farmers’ markets. Interestingly, these Y1+Y2 gardeners were over five times more likely to have gardened during childhood compared to non-gardeners. 

To the authors’ knowledge, this is the first report to examine the association between gardening experience and FV intake over multiple years in a population of college students. Intriguingly, despite the observational nature of this investigation, findings are largely consistent with those of long-term, intervention-based studies among children. For instance, at the end of the second year those who reported gardening during both academic years (Y1+Y2 gardeners) reported an average FV intake 0.6 CE/day higher than non-gardeners. The magnitude of this difference was remarkably similar to that observed by Wang et al. and Castro et al. at the conclusion of their respective two-year program [21]. Specifically, the former group observed a net between-group difference of 0.8 cups/day, while the latter group observed a within-group difference of approximately 0.7 cups/day. Collectively, these data provide several unique lines of evidence to support the association between long-term gardening experience and higher FV intake. Considering estimates for average FV intake among children and young adults are typically between 2 and 3 cups/day [3], it is reasonable to interpret these differences as clinically meaningful and sufficient for the advancement of research in this area. 

The present findings also provide insight into how change in gardening experience within the study period influences outcomes, an area that remains relatively unexplored in the literature. To our knowledge, only the study by Wang et al. has utilized a similar approach through the analysis of data collected after both the first and second year of the study [21]. Comparison of results yields several noteworthy themes. First, the continuation of gardening experience may offer little, if any, additional benefit beyond a certain point. This concept was originally proposed by Wang et al. after the authors observed a significant increase in FV intake after the first year of the program, but no further change after the second year. Likewise, the difference in FV intake between Y1+Y2 gardeners and non-gardeners was nearly the same at Y2 as it was at Y1 (0.6 CE/day and 0.7 CE/day, respectively). Although this may lead some to suggest that gardening experience beyond a certain point may not be worthwhile for the promotion of FV intake, evidence from both studies underscores the importance of continued gardening experience for sustained improvement in FV intake. For example, in the study by Wang et al., students who attended a school with high program exposure in the first year but low program exposure in the second year had a decrease in FV intake of nearly one serving per day from year one to year two. In our study, both Y1 only gardeners and Y1+Y2 gardeners reported higher FV intake than non-gardeners at Y1, but only Y1+Y2 gardeners reported a higher FV intake at Y2. 

Our findings related to FV intake among students who began gardening in year 2 (Y2 only gardeners) were somewhat unexpected. Despite reports of gardening experience during the preceding year, these students did not differ from non-gardeners in terms of Y2 FV intake. Although this is consistent with several reports in which gardening experience was limited to a single year, this observation may be a reflection of the manner in which gardening experience was assessed. At each time point, students were dichotomized based on self-reported gardening experience, but the specific duration, frequency, and degree of this experience could not be accounted for. Hence, although both Y1+Y2 gardeners and Y2 only gardeners reported gardening during the second year, Y2 only gardeners may have gardened for a shorter period within the year or on a less frequent basis than Y1+Y2 gardeners.

With respect to secondary outcomes, the observation that Y1+Y2 gardeners and non-gardeners significantly differ in FV-related attitudes and behaviors is meaningful on two levels. Since self-reported measures of dietary intake are prone to estimation errors [30], this provides an additional line of evidence to suggest that these groups differ with respect to FV intake. Indeed, similar metrics have been used in conjunction with FV intake to assess the effectiveness of gardening interventions, including several of those mentioned herein [9,13,21]. This finding is also significant in that it provides insight into how gardening experience may translate to higher FV intake. While some investigators have speculated that gardening-related improvements in FV intake are simply due to increased access to FV, our findings indicate gardening may impact other behaviors associated with greater FV intake. For example, the observation that Y1+Y2 gardeners shop at farmers markets and choose local foods more often than non-gardeners suggests that these students have developed unique priorities and motivations for purchasing FV. Regarding the other secondary outcome, the greater likelihood for Y+Y2 gardeners to have gardened during childhood, builds upon a central conclusion from our prior study. Previously we concluded that childhood gardening experience may be a key factor in the association between gardening experience and FV intake later in life. Here, we explore a potential underlying mechanism by specifically examining the relationship between childhood gardening experience and college gardening experience. Findings suggest that childhood gardening experience may represent an important foundation upon which students build the willingness or desire to maintain gardening experience while in college.

Interpretation of these findings warrants consideration of this study’s limitations, most of which stem from the observational design. For example, despite controlling for several established confounders, our ability to truly isolate the effect of gardening experience was limited in the absence of a controlled experimental setting. Likewise, our ability to infer causality is limited, since students who consume more FV than their peers may inherently be more likely to garden. With respect to the assessment of gardening experience in the past year, we dichotomized students based on the presence or absence of gardening experience, but did not further differentiate by degree of exposure among those with gardening experience. Although we were able to examine this in our previous study, accounting for a variety of unique patterns of experience over two full years was not feasible for this analysis. As mentioned previously, gardening experience just prior to the first year of college was not factored into the analysis. Therefore, it is possible that some students who reported gardening experience at Y1 (i.e., Y1 only gardeners and Y1+Y2 only gardeners) had consecutive gardening experience greater than one year at this time. Finally, because the parent study was designed to select for students at-risk for poor health behaviors while in college, this sub-sample was comprised of students who did not meet national recommendations for average daily FV intake at the time of enrollment. Therefore, if gardening experience is associated with higher FV intake as our work suggests, then students with gardening experience may have been underrepresented in this sample. 

This investigation has several notable strengths. As one of the few long-term studies to date, this investigation spanned a two-year period and utilized follow-up data after each year. We provided a more in-depth evaluation of outcomes compared to many previous reports through the analysis of full, partial, and non-exposures over the study period. In the case of Y1+Y2 gardeners and non-gardeners, the Y1 follow-up provided us with a mid-point assessment to examine how FV intake may change over the course of multi-year exposures. Additionally, the observational design of this study enabled us to consider all forms of gardening experience rather than what would be prescribed by a single intervention. Another notable strength was the inclusion of FV-related attitudes and behaviors. In addition to augmenting measures of self-reported FV intake, this allowed us to identify potential underlying mechanisms to further understand the association of interest. Finally, this study utilized a large, geographically diverse sample of nearly 600 students from institutions across the U.S.

Future studies in this area should continue to explore gardening experience over multiple years or growing seasons while accounting for the intensity of exposure across the study period. In the case of further observational work, this may require more regular follow-up and/or the development of new instruments to capture gardening experience over shorter intervals, such as monthly, seasonally, or by semester. Intervention studies should utilize multiple experimental groups with varying degrees of intervention exposure in addition to a non-gardening control group. Experimental work in this area should also assess non-intervention-related gardening experience as a study outcome, ideally through long-term follow-up. With respect to the assessment of FV intake, future studies should couple self-reported measures of FV intake with objective biomarkers, such as skin carotenoid status via resonance Raman spectroscopy. In addition to mitigating potential inaccuracies stemming from recall bias, this would provide a more standardized metric to enhance comparisons of results between studies. Finally, future research may benefit from the assessment of key behaviors or behavioral constructs related to FV intake to provide a more intricate, mechanistic understanding of the association of interest. 

## 5. Conclusions

Collectively, findings from this study suggest that gardening may be a promising strategy for the promotion of FV consumption among college students. While isolated experiences may be effective in this respect, consistent experience from year to year appears to be critical for sustained benefits over the long-term. Colleges and universities should consider developing gardening programs that foster reoccurring gardening experiences throughout an individual’s time as a student to promote student health and wellness. Such efforts may be particularly effective in parallel with grade school gardening programs or other initiatives to establish gardening experience earlier in life. 

## Figures and Tables

**Table 1:** Participant characteristics. Distribution of the overall sample (*N* = 593) across pertinent demographic characteristics, and university locations.

Participant Characteristics		*N* (%)
Sex	Male	178 (30.0%)
	Female	415 (70.0%)
Age (at Y1)	18	183 (30.9%)
	19	398 (67.1%)
	20	8 (1.3%)
	≥21	4 (0.7%)
Race/Ethnicity	White	289 (48.7%)
	Black	75 (12.6%)
	Hispanic/Latino	66 (11.1%)
	Other (including biracial)	162 (27.3%)
State	Alabama	27 (4.6%)
	Florida	199 (33.6%)
	Kansas	67 (11.3%)
	Maine	80 (13.5%)
	New York	88 (14.8%)
	South Dakota	28 (4.7%)
	Tennessee	61 (10.3%)
	West Virginia	43 (7.3%)
Total		593

**Table 2:** Prevalence of college gardening experience by participant characteristics (*N* = 593).

Participant Characteristics		Y1 Gardening Experience			Y2 Gardening Experience			Cumulative Gardening Experience				
		Yes	No	*p* Value	Yes	No	*p* Value	None	Y1 Only	Y2 Only	Y1+Y2	*p* Value
Sex	Male	46 (25.8%)	132 (74.2%)	0.13	45 (25.3%)	133 (74.7%)	0.19	114 (64.0%)	19 (10.7%)	18 (10.1%)	27 (15.2%)	0.28
	Female	87 (21.0%)	328 (79.0%)		84 (20.2%)	331 (79.8%)		284 (68.4%)	47 (11.3%)	44 (10.6%)	40 (9.6%)	
Race/Ethnicity	White	83 (28.7%)	206 (71.3%)	<0.01	73 (25.3%)	216 (74.7%)	0.06	178 (61.6%)	38 (13.1%)	28 (9.7%)	45 (15.6%)	0.02
	Black	9 (12.0%)	66 (88.0%)		12 (16.0%)	63 (84.0%)		58 (77.3%)	5 (6.7%)	8 (10.7%)	4 (5.3%)	
	Hispanic/Latino	10 (15.2%)	56 (84.8%)		10 (15.2%)	56 (84.8%)		50 (75.8%)	6 (9.1%)	6 (9.1%)	4 (6.1%)	
	Other	30 (18.5%)	132 (81.5%)		33 (20.4%)	129 (79.6%)		112 (69.1%)	17 (10.5%)	20 (12.3%)	13 (8%)	
State	Alabama	4 (14.8%)	23 (85.2%)	<0.001	6 (22.2%)	21 (77.8%)	0.01	18 (66.7%)	3 (11.1%)	5 (18.5%)	1 (3.7%)	<0.001
	Florida	31 (15.6%)	168 (84.4%)		37 (18.6%)	162 (81.4%)		143 (71.9%)	19 (9.5%)	25 (12.6%)	12 (6.0%)	
	Kansas	25 (37.3%)	42 (62.7%)		17 (25.4%)	50 (74.6%)		38 (56.7%)	12 (17.9%)	4 (6.0%)	13 (19.4%)	
	Maine	24 (30.0%)	56 (70.0%)		27 (33.8%)	53 (66.3%)		45 (56.3%)	8 (10.0%)	11 (13.8%)	16 (20.0%)	
	New York	13 (14.8%)	75 (85.2%)		14 (15.9%)	74 (84.1%)		67 (76.1%)	7 (8.0%)	8 (9.1%)	6 (6.8%)	
	South Dakota	14 (50.0%)	14 (50.0%)		11 (39.3%)	17 (60.7%)		11 (39.3%)	6 (21.4%)	3 (10.7%)	8 (28.6%)	
	Tennessee	12 (19.7%)	49 (80.3%)		8 (13.1%)	53 (86.9%)		46 (75.4%)	7 (11.5%)	3 (4.9%)	5 (8.2%)	
	West Virginia	10 (23.3%)	33 (76.7%)		9 (20.9%)	34 (79.1%)		30 (69.8%)	4 (9.3%)	3 (7.0%)	6 (14.0%)	
Total		133 (22.4%)	460 (77.6%)		129 (21.8%)	464 (78.2%)		398 (67.1%)	66 (11.1%)	62 (10.5%)	67 (11.3%)	

Significance of *p* < 0.05 was selected to determine within-group differences for each characteristic.

**Table 3:** FV-related attitudes and behaviors among Y1+Y2 Gardeners.

Item	Survey of Origin	Y1		Y2	
		OR (95% CI)	*p* Value	OR (95% CI)	*p* Value
How often do you eat locally grown foods?	Green Eating [25]	4.20 (2.54, 6.96)	< 0.001	3.22 (1.95, 5.30)	< 0.001
When in season, how often do you shop at farmer’s markets?	Green Eating [25]	3.91 (2.40, 6.39)	< 0.001	4.66 (2.84, 7.63)	< 0.001
How often do you choose foods that are certified organic?	Green Eating [25]	1.86 (1.15, 3.01)	0.114	1.40 (0.87, 2.25)	0.171
How often do you tell yourself that fruits and vegetables should be included in every meal?	Meal Planning [26]	1.58 (1.09, 2.75)	0.063	1.64 (1.02, 2.66)	0.043
How often do you purposefully add vegetables to meals and snacks?	Meal Planning [26]	1.92 (1.19, 3.11)	0.008	1.41 (0.88, 2.27)	0.157
In a normal week, how often do you prepare and cook a main meal from basic ingredients, for example, making lasagna starting with ground beef and tomato sauce?	Cooking [27]	2.19 (1.34, 3.58)	0.002	1.11 (0.70, 1.79)	0.647
How confident do you feel about being able to cook from basic ingredients?	Cooking [27]	1.95 (1.21, 3.14)	0.006	1.81 (1.11, 2.94)	0.017

The seven items used to assess FV-related attitudes and behaviors and the surveys from which they were selected are listed. Responses of Y1+Y2 gardeners were analyzed at each time point using non-gardeners as a reference group. Hence, corresponding odds ratios can be interpreted as the odds of a Y1+Y2 gardener choosing a higher level of agreement compared to a non-gardener at that time. The model controlled for university, BMI, sex, and race/ethnicity. Level of significance set at *p* < 0.05.

## Supplementary Materials

The following are available online at https://www.mdpi.com/2072-6643/11/9/2088/s1, Table S1: Between-group differences in college gardening experience by race/ethnicity, Table S2: Between-group differences in college gardening experience by university location.
