# Supplementary material for: A Multi-Year Examination of Gardening Experience and Fruit and Vegetable Intake During College
Source: Nutrients. 2019 Sep 4;11(9):2088. doi: 10.3390/nu11092088 (PMC6770305; doi:10.3390/nu11092088)
Supplement: Supplementary file 1 [file nutrients-11-02088-s001.pdf]

Supplementary Table S1. Between-group differences in college gardening experience by race/ethnicity.

	Prevalence (Within-Group)			
	**White[a]**	**Black[b]**	**Hispanic/Latino[c]**	**Other[d]**
**Y1 Gardening Experience**	28.7%[b,c]	12%[a]	15.2%[a]	18.5%
**Y2 Gardening Experience**	25.3%	16.0%	15.2%	20.4%
Cumulative Gardening Experience				
**Non-Gardeners**	61.6%[b,c]	77.3%[a]	75.8%[a]	69.1%
**Y1 Only Gardeners**	13.1%[b]	6.7%[a]	9.1%	10.5%
**Y2 Only Gardeners**	9.7%	10.7%	9.1%	12.3%
**Y1+Y2 Gardeners**	15.6%[b,c]	5.3%[a]	6.1%[a]	8.0%

Supplementary Table S2. Between-group differences in college gardening experience by university location.

	Prevalence (Within-Group)							
	**Alabama[a]**	**Florida[b]**	**Kansas[c]**	**Maine[d]**	**New York[e]**	**South Dakota[f]**	**Tennessee[g]**	**West Virginia[h]**
**Y1 Gardening Experience**	14.8%[c,d,f]	15.6%[c,d,f]	37.3%[a,b,e,g]	30%[a,b,e]	14.8%[c,d,f]	50%[a,b,e,g,h]	19.7%[c,f]	23.3%[f]
**Y2 Gardening Experience**	22.2%[f]	18.6%[d,f]	25.4%	33.8%[b,e,g]	15.9%[d,f]	39.3%[a,b,e,g,h]	13.1%[d,f]	20.9%
Cumulative Gardening Experience								
**Non-Gardeners**	66.7%	71.9%[f]	56.7%	56.3%	76.1%[f]	39.3%[b,e,g,h]	75.4%[f]	69.8%[f]
**Y1 Only Gardeners**	11.1%	9.5%	17.9%	10.0%	8.0%[f]	21.4%	11.5%	9.3%
**Y2 Only Gardeners**	18.5%	12.6%	6.0%	13.8%	9.1%	10.7%	4.9%	7.0%
**Y1+Y2 Gardeners**	3.7%[c,d,f]	6%[c,d,f]	19.4%[a,b,e,g]	20%[a,b,e,g]	6.8%[c]	28.6%[a,b,e,g]	8.2%[c,d,f]	14%

*Each letter in superscript corresponds to the group indicated in the top row. Values followed by a superscript(s) differ significantly ($p < 0.05$) from that of the corresponding group(s).
